# FrpA is the outer membrane piscibactin transporter in *Vibrio anguillarum*: structural elements in synthetic piscibactin analogues required for transport

**DOI:** 10.1007/s00775-021-01916-1

**Published:** 2021-11-18

**Authors:** Marta A. Lages, M. Carmen de la Fuente, Lucía Ageitos, Diana Martínez-Matamoros, Jaime Rodríguez, Miguel Balado, Carlos Jiménez, Manuel L. Lemos

**Affiliations:** 1 Departamento de Microbiología y Parasitología, Instituto de Acuicultura, Universidade de Santiago de Compostela, 15782 Santiago de Compostela, Spain; 2 Departamento de Química, Facultade de Ciencias, Centro de Investigacións Científicas Avanzadas (CICA), Universidade da Coruña, 15071 A Coruña, Spain

**Keywords:** *Vibrio anguillarum*, *Photobacteriumdamselae* subsp. *piscicida*, *Siderophores*, *Piscibactin*, Fe(III)-siderophore transporter

## Abstract

Piscibactin (Pcb) is a labile siderophore widespread among *Vibrionaceae*. Its production is a major virulence factor of some fish pathogens such as *Photobacterium damselae* subsp. *piscicida* and *Vibrio anguillarum*. Although FrpA was previously suggested as the putative outer membrane transporter (OMT) for ferri-piscibactin, its role in piscibactin uptake was never demonstrated. In this work, we generated mutants of *V. anguillarum* defective in FrpA and analyzed their ability to use piscibactin as iron source. The results showed that inactivation of *frpA* completely disables piscibactin utilization, and the original phenotype could be restored by gene complementation, confirming that FrpA is the OMT that mediates ferri-Pcb uptake. Additionally, the ability of several Pcb thiazole analogues, with different configurations at positions 9, 10, and 13, to be internalized through FrpA, was evaluated measuring their ability to promote growth under iron deficiency of several indicator strains. The results showed that while those analogues with a thiazole ring maintain almost the same activity as Pcb, the maintenance of the hydroxyl group present in natural piscibactin configuration at position C-13 is crucial for Fe^3+^ chelation and, in consequence, for the recognition of the ferri-siderophore by the cognate OMT. All these findings allowed us to propose a Pcb analogue as a good candidate to vectorize antimicrobial compounds, through the Trojan horse strategy, to develop novel compounds against bacterial fish diseases.

**Graphical abstract:**

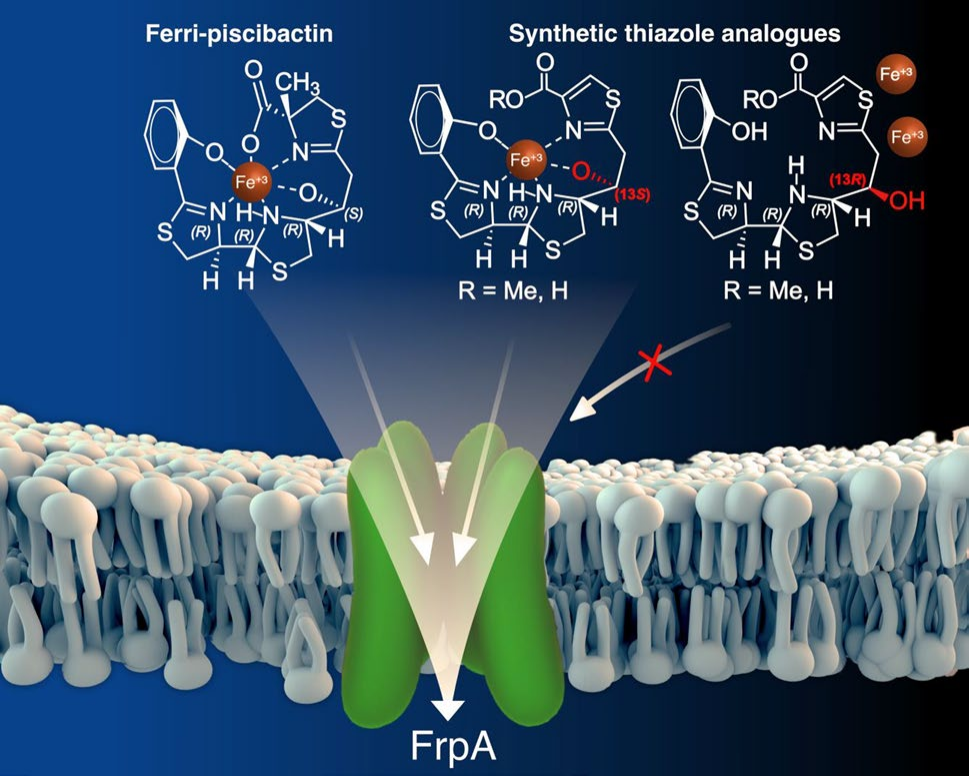

**Supplementary Information:**

The online version contains supplementary material available at 10.1007/s00775-021-01916-1.

## Introduction

Bacteria belonging to *Vibrionaceae* family are present as free-living organisms in seawater, marine sediments, or colonizing marine organisms as symbionts. In addition, several pathogenic species for animals and humans are members of this family [1]. The spread of *Vibrio* spp. is favored by climate change and global seawater warming, which explains the increasing incidence of vibriosis in marine animals [2]. The emergence of novel diseases leads to a bottleneck effect that blocks the aquaculture sector from higher profit [3, 4]. Although vaccination was shown as the most efficient way to minimize the incidence of infectious diseases [5], once outbreaks occur, the use of antibacterial compounds such as antibiotics is unavoidable. Unfortunately, their use increases the risk of drug resistance appearance in microbiota associated with fish farms and could also have adverse effects on consumers [6]. In this context, it is necessary the search of novel safe antimicrobials that can be effective against a wide range of bacterial pathogens.

The development of disease is closely associated with environmental signals that induce the expression of virulence factors [7]. Iron is essential for the survival and growth of almost all bacteria, but its availability is limited in the environment and within the host fluids [8]. In response to iron starvation, several pathogens have developed specific iron uptake mechanisms such as the synthesis and secretion of siderophores [9]. These iron chelators are secondary metabolites produced by most pathogenic bacteria to overcome iron starvation. Once the siderophore binds iron(III), the ferri-siderophore complex is acquired by bacteria through specific transporters [10]. Among the strategies aimed at developing novel antimicrobials, a promising approach called “Trojan Horse” consists in the use of siderophores conjugated to antimicrobial molecules which lead to a significantly increased antimicrobial activity, since they are using the high specific siderophore transport system to be internalized [11, 12]. Using this strategy, Cefiderocol, a catechol-substituted siderophore cephalosporin, was recently approved for the treatment of aerobic Gram-negative bacteria infections in humans [13].

Piscibactin (Pcb, **1**) is a siderophore encoded by *irp* genes located in a high-pathogenicity island named *irp*-HPI [14] and its production is a major virulence factor of some fish pathogens such as *Photobacterium damselae* subsp. *piscicida* (*Pdp*) [15] and *Vibrio anguillarum* (*Va*) [16], two of the most devastating bacterial pathogens in aquaculture worldwide. Notably, *irp*-HPI is widespread among *Vibrionaceae* [17]*.* A possible intermediate of Pcb, prepiscibactin (**3**) was also isolated from *Pdp* [15]. Thus, the piscibactin system could constitute a good candidate to vectorize antimicrobial compounds that could be used to develop novel antibacterials against the main bacterial fish diseases affecting aquaculture. Notably, the structure of Pcb (**1**) is very similar to that of yersiniabactin (**2**), the siderophore produced by some *Yersina* species such as *Y. pestis* and *Y. enterocolitica*, causative agents of severe enteric disorders in humans [18].

**Fig. 1:**
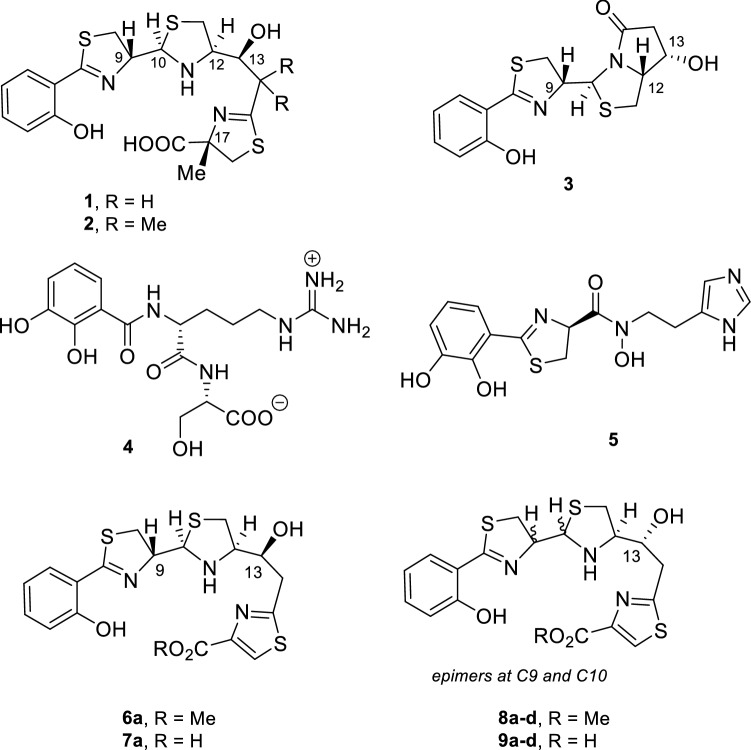
Structures of the three siderophores, piscibactin (**1**), vanchrobactin (**4**), and anguibactin (**5**), involved in the iron (III) uptake in *Vibrio anguillarum*, yersiniabactin (**2**), prepiscibactin (**3**), and some of the thiazole-Pcb analogues, compounds **6**–**9**, used in this work (the synthesis of these analogues will be published elsewhere)

**Fig. 2:**
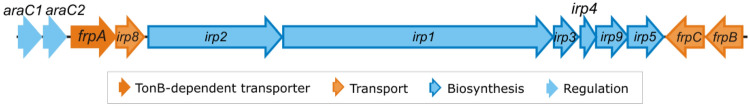
Genetic map of *irp* gene cluster encoding piscibactin (Pcb) biosynthesis and transport

Besides piscibactin (**1**), two other siderophores have been described in *V. anguillarum*: vanchrobactin (**4**)[19] and anguibactin (**5**)[20] (Fig. 1). The high-pathogenicity island *irp*-HPI encodes functions related to piscibactin biosynthesis (*irp123459*), ferri-piscibactin transport (*frpABC*) and regulation of the system (*araC1* and *araC2*) (Fig. 2) [14, 15]. A synthesis pathway was proposed based on the domain organization of biosynthetic enzymes [14]. Although according to amino acid sequence similarities the outer membrane protein FrpA, encoded by *frpA* gene in *irp*-HPI, should be the outer membrane transporter (OMT) of Pcb (**1**), its actual role in the ferric-siderophore internalization was never demonstrated. On the other hand, to rationally design vectors that exploit the “Trojan horse” strategy based on Pcb-conjugates, the transporter(s) involved in Pcb uptake must be characterized.

In this work, *V. anguillarum* was used as a model to study the role of FrpA in the uptake of the siderophore Pcb (**1**) by generating defective mutants of *frpA* gene. The results confirmed that FrpA is the outer membrane transporter that mediates ferri-Pcb uptake. Furthermore, the evaluation of the ability of several thiazole-Pcb analogues (**6**–**9**) to act as Fe^3+^ chelators and to be internalized via FrpA allowed us to determine structure–activity relationships (SAR) that would be essential for recognition of ferri-Pcb complex by its cognate transporter FrpA. The obtained results will be valuable for the preparation of conjugates, such as fluorescent probes or novel antimicrobials against piscibactin-synthesizing bacterial pathogens.

## Materials and methods

### Bacterial strains, plasmids, and media

Bacterial strains and plasmids used in this work are listed in Table S1. *V. anguillarum* and *P. damselae* subsp. *piscicida* strains were grown at 25 °C or 15 °C in Tryptic soy agar (TSA-1) or broth (TSB-1) supplemented with 1% NaCl. *Escherichia coli* strains were grown at 37 °C in Luria–Bertani (LB) agar or broth. When required, the medium was supplemented with antibiotics at the correspondent final concentration of: ampicillin sodium salt (Amp) at 60 µg mL^−1^ or 100 µg mL^−1^, kanamycin (Kan) at 50 µg mL^−1^, and gentamicin (Gen) at 15 µg mL^−1^.

### Construction of *frpA* defective mutants by allelic exchange

In frame deletions of the gene *frpA* were constructed by allelic exchange in *V. anguillarum* strains RV22 ∆*vabF* (unable to synthesize vanchrobactin) and RV22 ∆*vabD* (unable to synthesize both vanchrobactin and piscibactin). The flanking regions of *frpA* were amplified by PCR and cloned into the high copy vector pWKS30. The construction was ligated into the suicide vector pNidKan [21]. The resulted plasmid was conjugated with the previously mentioned strains and the selection was performed based on kanamycin resistance. After a second recombination event, the mutant strains were selected based on sucrose resistance (15%), and the consequent plasmid loss was confirmed by screening the growth on kanamycin and ampicillin plates. Subsequently, a PCR was performed to confirm the allelic exchange. This led to the generation of the mutant strains RV22 ∆*vabF∆frpA* and RV22 ∆*vabD*∆*frpA*. For the complementation of the gene, *frpA* was amplified by PCR and cloned into the vector pSEVA651 in *E. coli* S17-1 λpir. The plasmid was mobilized into the mutant strains to restore the original phenotypes. The oligonucleotides used are listed in Table S2.

### Growth ability and siderophore production under iron-limiting conditions

Overnight cultures of *V. anguillarum* RV22 ∆*vabF*, RV22 ∆*vabF∆frpA*, RV22 ∆*vabD*, and RV22 ∆*vabD∆frpA* were made in TSB-1. The OD_600_ was adjusted to 0.5 and a 1:50 dilution was inoculated in 5 mL of CM9 medium. Each culture was supplemented with 10 µM FeCl_3_, for iron excess conditions or with 50 or 75 µM 2,2ʹ-dipyridyl to achieve the iron-deficient conditions. The cultures were incubated at 15 °C, with shaking at 120 rpm. After 48 h, the final growth was recorded in a spectrophotometer (Hitachi). Additionally, when the cultures reached an OD_600_ ≈ 0.8, they were centrifuged at 10,000 rpm and the supernatant was used to perform a chrome azurol-S (CAS) liquid assay [22]. The supernatant was mixed 1:1 with the CAS reagent. After 15 min of incubation at room temperature, the A_630_ was measured in a spectrophotometer (Hitachi).

### Isolation of Piscibactin-Fe(III) complex from *Vibrio anguillarum* RV22 Δ*vabF*

Using the procedure describes by Souto et al. [15], the isolation of piscibactin-Fe(III) was carried out as follows: 1 L of centrifuged cell-free culture broth of *Vibrio anguillarum* RV22 Δ*vabF* was treated with 17 mg of FeCl_3_**·**6H_2_O and then concentrated in vacuo to 0.30 L. The sample was passed through four OASIS^®^ HLB cartridges (35 cm^3^, 6 g), eluted with 30 mL of the following mixtures of H_2_O and CH_3_CN: 1:0, 7:3, 1:1, 3:7, 0:1, and the fraction eluted with a mixture of H_2_O and CH_3_CN (1:1) was collected. This fraction was concentrated under vacuum to about 5 mL under reduced pressure and submitted to RP-HPLC. Finally, separation was achieved using a Discovery^®^ HS F5 (100 × 4.6 mm, 5 μm) column (Supelco) with a 5 min gradient from 10 to 20% of CH_3_CN in H_2_O, a 10 min isocratic period at 20% of CH_3_CN in H_2_O, a 5 min gradient from 20 to 100% CH_3_CN in H_2_O and finally a 5 min isocratic period at 100% of CH_3_CN at a flow rate of 1 mL/min (UV detector operating at *λ* = 264 nm). Fractions containing piscibactin-Fe(III) complex (100% of CH_3_CN in H_2_O, *R*_*t*_ = 20 min) were pooled and dried under vacuum. This procedure provided ca. 1 mg of piscibactin-Fe(III) complex.

### Cross-feeding assays

Cross-feeding assays were performed to analyze whether several *V. anguillarum* strains could use piscibactin as iron source. 1 mL of a TSB-1 culture at an OD_600_ ≈ 0.8 of the indicator strains, RV22 ∆*vabF* and RV22 ∆*vabF∆frpA*, was inoculated into 20 mL of CM9 minimal medium containing 0.8% agarose and supplemented with 90 µM 2,2ʹ-dipyridyl and poured onto plates. The strains to be tested for piscibactin production RV22 ∆*vabF* (piscibactin producer), RV22 ∆*vabD* (unable to produce piscibactin and vanchrobactin), and RV22 ∆*vabF∆frpA* were cultured in TSA-1 plates supplemented with 50 µM 2,2ʹ-dipyridyl, and a loopful of biomass was harvested and placed onto the surface of the plates previously inoculated with the indicator strain. After 48 h incubation at 25 °C, a growth halo around the tested strains indicated the utilization of the siderophore.

### Biological activity of piscibactin analogues

The biological activity of piscibactin analogues, **6**–**9**, was evaluated in 96-well microtiter plates, using 200 µL as final volume. To determine the lowest concentration that allows growth, piscibactin and each analogue were used at the final concentrations of 20, 10, 2, and 0.2 µM from a stock solution prepared with methanol:milliQ water (1:1). From an overnight culture of RV22 ∆*vabD*, RV22 ∆*vabD∆frpA* and *P. damselae* subsp. *piscicida*, whose OD_600_ was adjusted to 0.5, a final dilution of 1:20 and 1:40, respectively, was inoculated in CM9 media supplemented with 75 µM 2,2ʹ-dipyridyl. After the addition of piscibactin and the tested analogues at the suitable concentrations, the plate was incubated at 25 °C with shaking at 120 rpm. The growth (OD_600_) was recorded for 18 h in an iMACK Microplate reader (Bio-Rad). Media supplemented with ferric chloride (10 µM FeCl_3_), with the chelating agent 2,2ʹ-dipyridyl (75 µM), and non-inoculated media were used as controls. The assay corresponding to each condition (iron excess or iron deficiency) and each bacterial strain was performed in duplicate in each plate. Three independent experiments were performed, and the results shown are the mean of the values obtained.

### FrpA phylogenetic analysis

To analyze the diversity of piscibactin outer membrane receptor FrpA, we performed BlastN searches in the nucleotide collection (nr/nt) and whole-genome shotgun (wgs) NCBI databases using as a query *frpA* nucleotide sequence of *V. anguillarum* RV22. *frpA* homologs were downloaded from NCBI and aligned using MUSCLE (MEGA 6 suite). The phylogenetic tree was constructed by the neighbor-joining method with a bootstrap method of 1000 replicates using also MEGA 6 software.

## Results and discussion

### Inactivation of *frpA* disables Pcb utilization as iron source

**Fig. 3:**
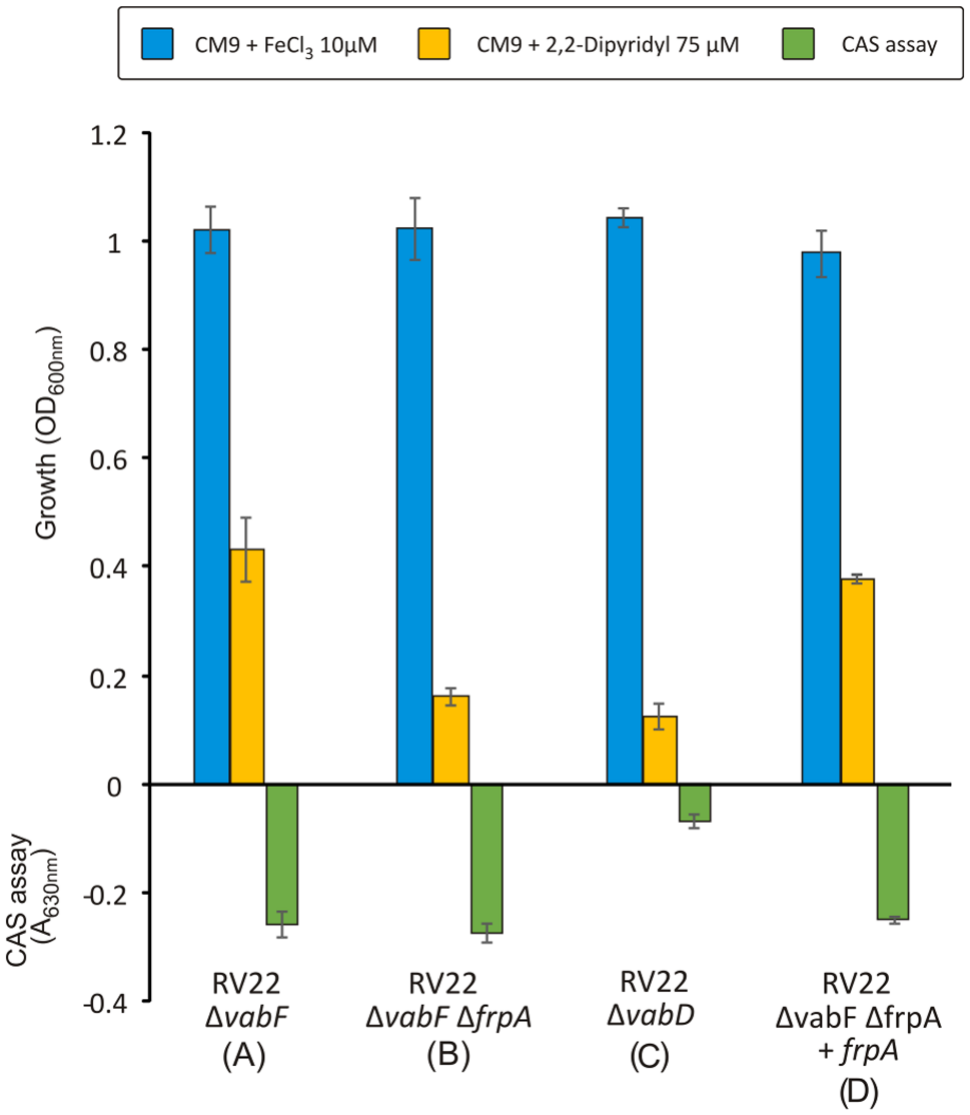
Growth under high or low iron conditions and siderophore production measured by CAS assay of parental strain (**A**) *V. anguillarum* RV22 *∆vabF*, its derivative ∆*frpA* mutant (**B**) (RV22 ∆*vabF∆frpA*), and control strain (**C**) (RV22 *∆vabD*) that is unable to synthetize any siderophore. The *frpA* complemented strain (**D**) was obtained by the introduction of the wild-type *frpA* gene, cloned in the plasmid pSEVA, into *V. anguillarum* ∆*frpA* mutant

The *irp*-HPI island contains genes (*frpABC*) that are predicted to encode the ferri-piscibactin uptake machinery (Fig. 2) [16]. Particularly, *frpA* gene would encode the putative outer membrane transporter of the ferri-piscibactin complex. To confirm the role of FrpA in Pcb uptake, an in-frame deletion mutant for this gene was obtained using *V. anguillarum* RV22 ∆*vabF* as parental strain*,* a strain that only synthesizes Pcb (**1**) and not vanchrobactin. The ability of the generated mutant to use Pcb (**1**) as iron source was first studied by its ability to grow under iron deficiency. Strain RV22 ∆*vabD*, a mutant unable to synthesize neither Pcb (**1**) nor vanchrobactin (**4**), and thus unable to grow under iron deficiency, was used as negative control. As shown in Fig. 3, while the parental strain RV22 ∆*vabF* could grow under high iron deficiency (CM9 containing 2,2´-dipyridyl at 75 µM), the *frpA* defective mutant (RV22 ∆*vabF∆frpA*) was totally impaired to grow under these conditions. Complementation of the *frpA* defective mutant with a copy of the wild-type gene restored the growth phenotype at the same levels as the parental strain (Fig. 3). These results clearly indicate that FrpA must act as the OMT for Pcb (**1**).

**Fig. 4:**
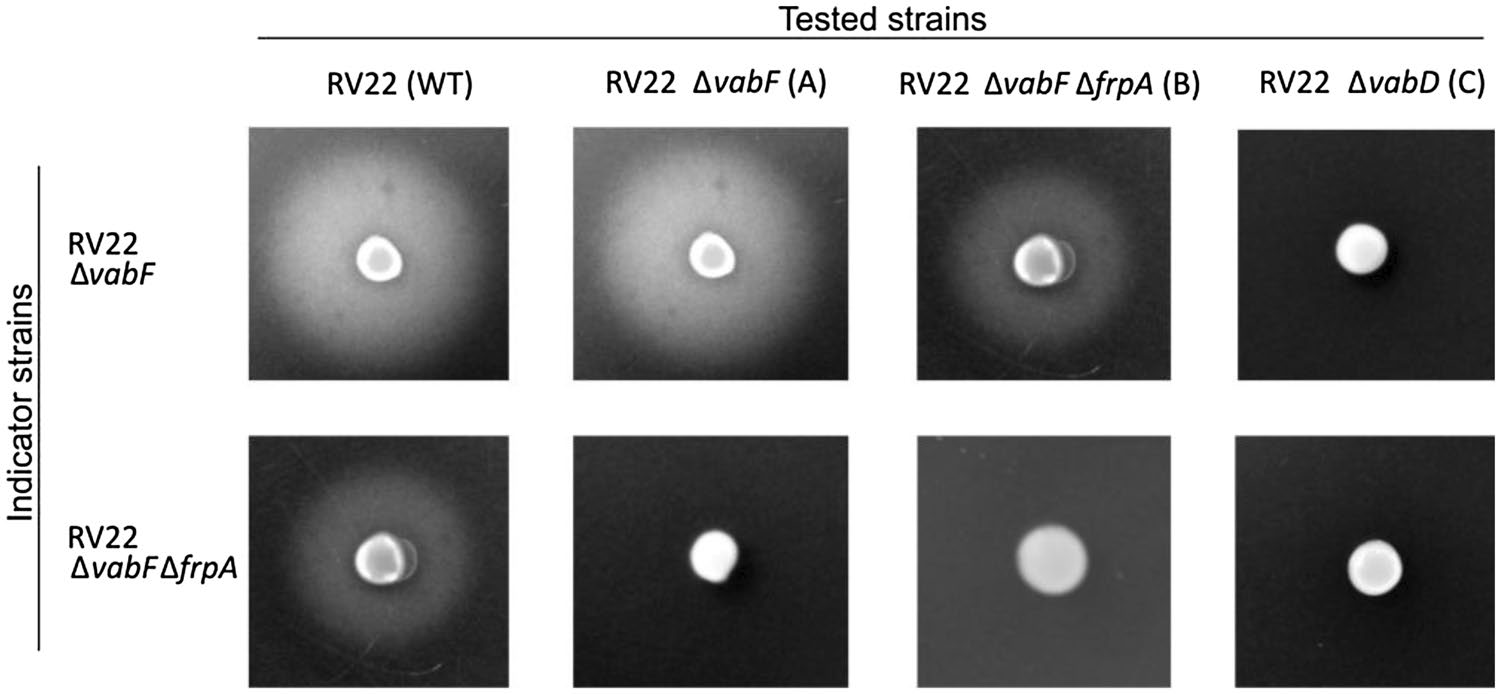
Cross-feeding assay to analyze the utilization of Pcb (**1**) by an FrpA mutant. RV22 ∆*vabF* [producing Pcb (**1**) but unable to produce vanchrobactin] and RV22 ∆*vabF∆frpA* [producing Pcb (**1**) but potentially unable to utilize it] were used as indicator strains. Wild-type strain RV22 (WT) and its derivative mutants RV22 ∆*vabF* (**A**), RV22 ∆*vabF∆frpA* (**B**) and RV22 ∆*vabD* (**C**) (unable to synthesize any siderophore), were used as tested strains. A growth halo of the indicator strains around the tested strains evidences that they can use the siderophore produced by the tested strains to grow under iron-limited conditions

In addition, the evaluation of siderophore activity in supernatants by the CAS assay showed that the mutant RV22 ∆*vabF∆frpA* produces the same siderophore levels as its parental strain RV22 ∆*vabF* (Fig. 3) and that it was able to cross-feed RV22 ∆*vabF* (Fig. 4). However, RV22 ∆*vabF∆frpA* could not be cross-fed by any piscibactin-producer strain, except by the wild-type RV22 due to the utilization of vanchrobactin. Thus, cross-feed assays confirm that the mutant defective in FrpA outer membrane transporter (RV22 ∆*vabF∆frpA*) was impaired to use Pcb (**1**) as iron source (Fig. 4).

The results obtained in the grow cross-feeding assays greatly suggest that FrpA has an active role in the ability of RV22 ∆*vabF* to grow in iron-limited conditions and that it must be involved in the internalization of ferri-piscibactin, acting as the OMT for this siderophore.

Outer membrane transporters of siderophores are generally considered to be quite specific for their cognate ligand [23]. However, some works identified siderophore transporters that can transport multiple related ligands [24–27]. One of the most versatile siderophore transporters described to date is FstC, the amonabactin OMT of *A. salmonicida* [27]. FstC mediates the uptake of the four natural amonabactin forms and also internalizes some biscatecholate amonabactin analogues with rather different chemical structures [27]. The successful use of siderophore uptake mechanisms to vectorize compounds depends on the selection of a versatile target transporter and the characterization of the structures of the siderophore required for its recognition by the cognate OMT. Thus, once demonstrated that FrpA is the outer membrane transporter for piscibactin, some piscibactin analogues were synthesized and analyzed for their ability to be internalized by FrpA.

### Evaluation of the siderophore activity of Pcb (1) and Pcb thiazole analogues 6–9 in *V. anguillarum*

Since the presence of the acid sensitive β-hydroxy-2,4-disubstituted thiazoline moiety [28] in Ga^+3^–Pcb complex (**1**-Ga^+3^) represented a challenge in our reported total synthesis for this compound [29], we have found that the substitution of this thiazoline ring for a thiazole ring that is less sensitive to acids, due to its aromatic character, facilitated the synthesis of analogues (manuscript in preparation).

**Fig. 5:**
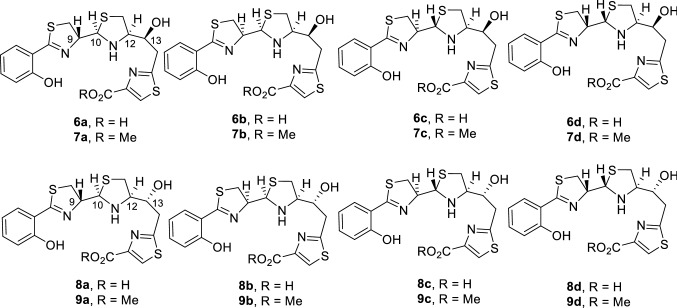
Structure of the synthetic Pcb thiazole analogues **6**–**9** (manuscript in preparation) evaluated in this work

In this way, two sets of Pcb analogues bearing a thiazole ring (**6**–**9**) but differing in the configuration of the hydroxy group at C-13 position (Fig. 5) were previously synthetized and then evaluated with the aim of determining some structural requirements for Pcb (**1**) recognition by its OMT protein FrpA and searching for a simplified Pcb analogue that keeps the siderophore activity.

**Fig. 6:**
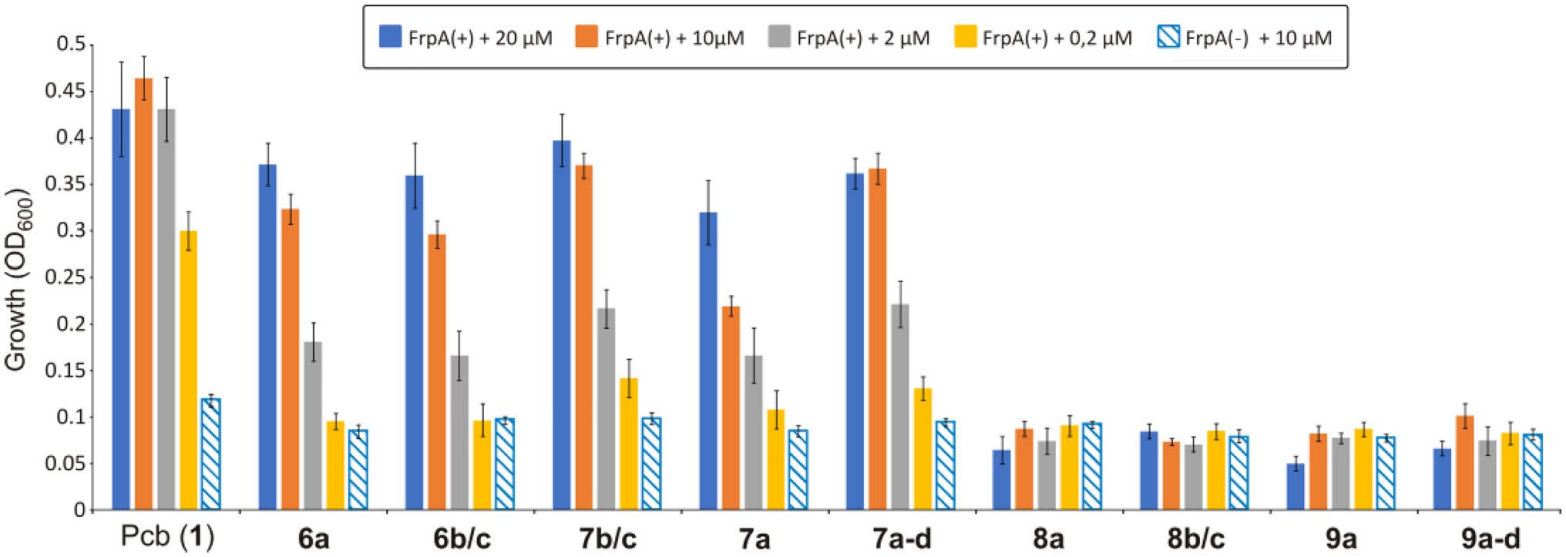
Biological activity (maximum growth achieved) of Pcb (**1**) and thiazole-Pcb analogues **6**–**9** measured by growth promotion of a *V. anguillarum* FrpA( +) strain, carrying a functional *frpA* gene (RV22 ∆*vabD*), or an FrpA(−) strain (RV22 ∆*vabD∆frpA*). The assays were performed in CM9 minimal medium supplemented with 75 µM 2,2ʹ-dipyridil and one of the compounds to be tested at concentrations between 0.2 and 20 µM. Error bars represent standard deviation

The biological activity of synthetic Pcb thiazole analogues **6**–**9** was compared to the natural Pcb (**1**) using growth promotion assays against FrpA( +) (RV22 ∆*vabD*) and FrpA(−) (RV22 ∆*vabD∆frpA*) strains (Fig. 6). Since inactivation of *vabD* abolishes siderophore production, both vanchrobactin and piscibactin [16], the growth achieved by these strains will be proportional to the bio-availability of the Pcb or analogue **6**–**9** tested. Pcb (**1**) efficiently promoted, in iron-limiting conditions, the growth of *V. anguillarum* RV22 ∆*vabD* mutant. For each concentration tested, Pcb (**1**) showed the highest siderophore activity of all compounds tested. As expected, Pcb (**1**) was unable to restore the growth of the FrpA(−) defective mutant, which confirms that FrpA mediates the iron uptake of Pcb (**1**) in *V. anguillarum* RV22 (Fig. 6). Notably, addition of Pcb (**1**) in the range 2–20 µM increased the maximum growth achieved (Fig. 6), being indistinguishable from that observed when ferric chloride was added to the medium. Results of growth promotion assays showed that all 13*S* thiazole-Pcb analogues, compounds **6** and **7**, stimulate the growth of FrpA( +) at all concentrations tested. However, they were less effective than endogenous Pcb (**1**). On the contrary, 13*R* thiazole analogues **8** and **9** with opposite configuration at position C-13 did not support growth of neither FrpA( +) nor FrpA( −) (Fig. 6).

Chrome azurol-S (CAS) liquid assay was used to assess the ability of each analogue to chelate iron [22]. Results showed that Pcb (**1**) and the 13*S* thiazole-Pcb analogues **6** and **7** gave a positive CAS test, which showed that these compounds (**1**, **6,** and **7**) chelate iron. By contrast, 13*R* thiazole-Pcb analogues **8** and **9** displayed a negative result in the CAS assay. These findings are in agreement with unsuccessful Ga^3+^ complexation attempts with the 13*R* acid thiazole analogues **8**. Additionally, although the mixture of thiazole-Pcb analogues **7b**,**c** exhibited a slight higher biological activity than their corresponding methyl esters **6b**,**c** and thiazole-Pcb analogue **7a**, the differences were not statistically significant. In the same way, similar activities were obtained for 13*S* thiazole-Pcb analogues **6** and **7** which suggest that the configuration at positions C-9 and C-10 has no major influence on the biological activity of these analogues. All results put together greatly suggest that the configuration of the hydroxyl group at C-13 is crucial for the chelation of Fe^3+^ and, in consequence, for the molecular recognition by the outer membrane transporter FrpA. Most notably, the siderophore activity showed by the 13*S* thiazole analogues **6** and **7** indicates that the substitution of the thiazoline ring for a thiazole ring and/or the presence of a methyl ester group instead of a carboxylic acid functionality does not affect to the recognition of Pcb analogues by FrpA. The possible presence of esterases during the uptake process could explain the similar biological activity of the methyl esters in relation to their corresponding acids. These results suggest that these groups do not constitute key structural features of the siderophore and likely they do not interact in a distinctive way with the recognition domain of the FrpA transporter.

Pcb (**1**) and yersiniabactin (**2**) share structural characteristics and their labile nature is a disadvantage in terms of chemical synthesis and manipulation of the molecule [15, 30]. Pcb (**1**) can be stabilized by chelation with iron(III) or gallium(III) [15]. As previously described, Pcb (**1**) chelates gallium(III) through the three nitrogens and the three oxygens present in the siderophore [15]. Our result showed that configuration at position C-13 is crucial for metal chelation and, consequently, its alteration disables the biological activity of the siderophore.

Taking into account that the substitution of the thiazoline ring in Pcb (**1**) for a thiazole ring facilitated the synthesis of Pcb analogues and the fact that their methyl ester derivatives efficiently promote the growth of FrpA( +) bacterial strains (Fig. 6), these type of analogues could be good candidates to be used as vectors for future conjugation with antimicrobials or fluorescent probes following the “Trojan horse strategy”.

### Phylogenetic analysis of *frpA* and siderophore activity in *P. damselae* subsp. *piscicida*

**Fig. 7:**
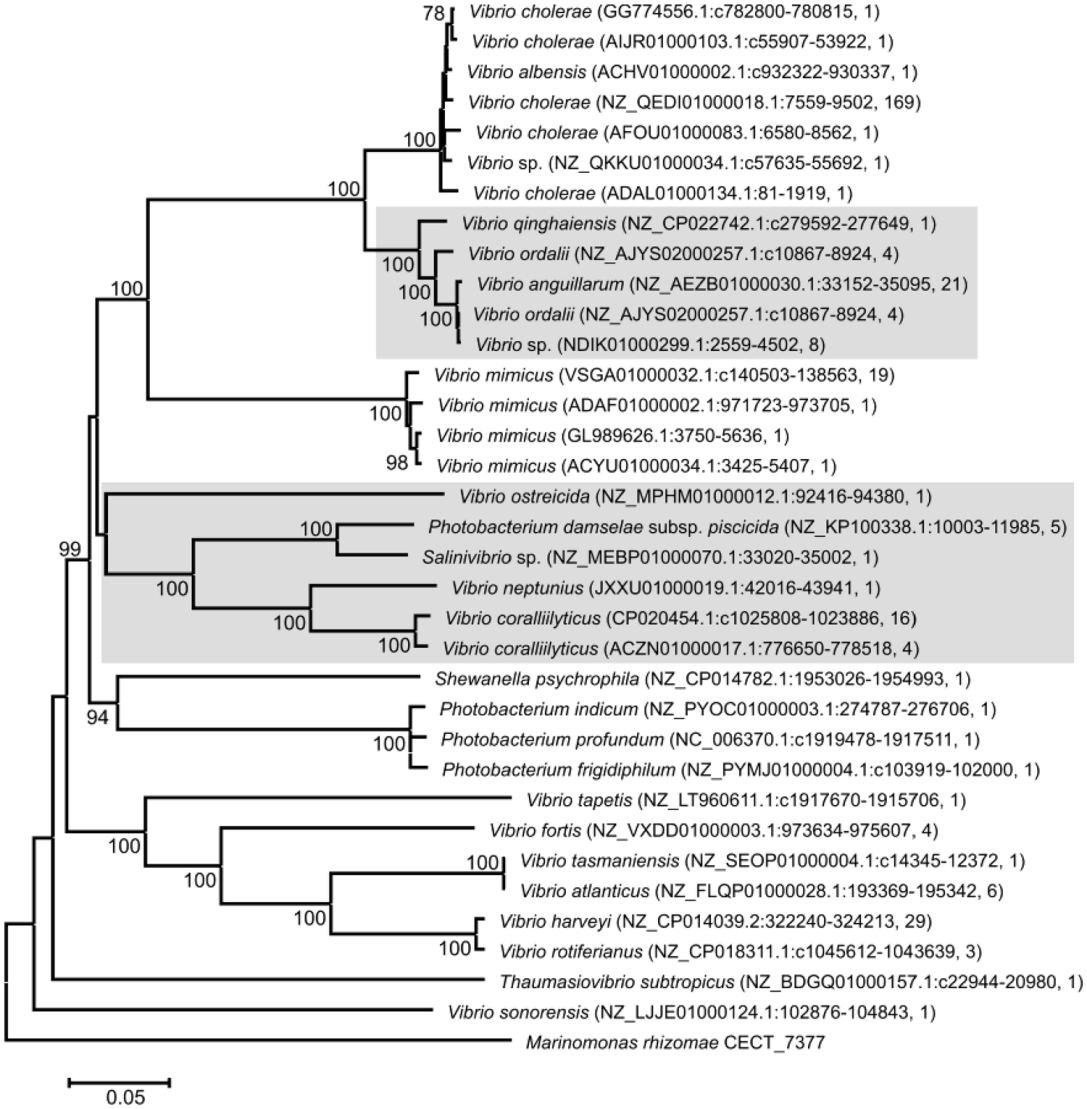
Phylogenetic tree of *frpA* gene homologues encoding the Pcb outer membrane transporter FrpA found in GenBank (black). Labels denote species name, accession number, and number of identical genes deposited in GenBank. The tree is drawn to scale and branch lengths denote nucleotide p-distance (p-distance method). Bootstrap values higher than 70% are shown next to the branches. All positions containing gaps and missing data were eliminated. There was a total of 611 positions in the final dataset

Piscibactin siderophore system is encoded by a genomic island (*irp*-HPI) that is widespread among *Vibrionaceae*, including species grouped in the Harveyi and Splendidus clades [16, 17, 21, 31]. Thus, some different versions (homologues) of the *frpA* gene encoding the piscibactin transporter FrpA are present in different bacterial species. A phylogenetic analysis with those *frpA* versions available in GenBank was performed. The resultant phylogenetic tree is shown in Fig. 7. The results showed that *frpA* is widespread not only in *Vibrionaceae*, but also it is present in many species of gamma-proteobacteria like *Shewanella*, *Marinomonas* and enterobacteria like *Xenorhabdus*, *Photorhabdus*, and *Providencia* with similarities higher than 62%. *V. anguillarum frpA* (*frpA*_*Vang*_) is clustered in a clade with *V. ordalii* and *V. qinghaiensis*. In addition, these *frpA* sequences are clustered with a bootstrap value of 100 with several *V. cholerae* sequences, which reflect a close relationship between the *frpA* homologues present in the genome of these bacteria.

The outer membrane transporter FrpA of *V. anguillarum* (FrpA_*Vang*_) shares a 66% homology identity and 81% similarity with the version of FrpA present in the genome of *P. damselae* subsp. *piscicida* (FrpA_*Pdp*_). Gene *frpA*_*Pdp*_ is clustered with *Vibrio* species generally related to bivalve molluscs infections such as *V. ostreicida*, *V. coralliilyticus*, and *V. neptunius* [32]*.*

**Fig. 8:**
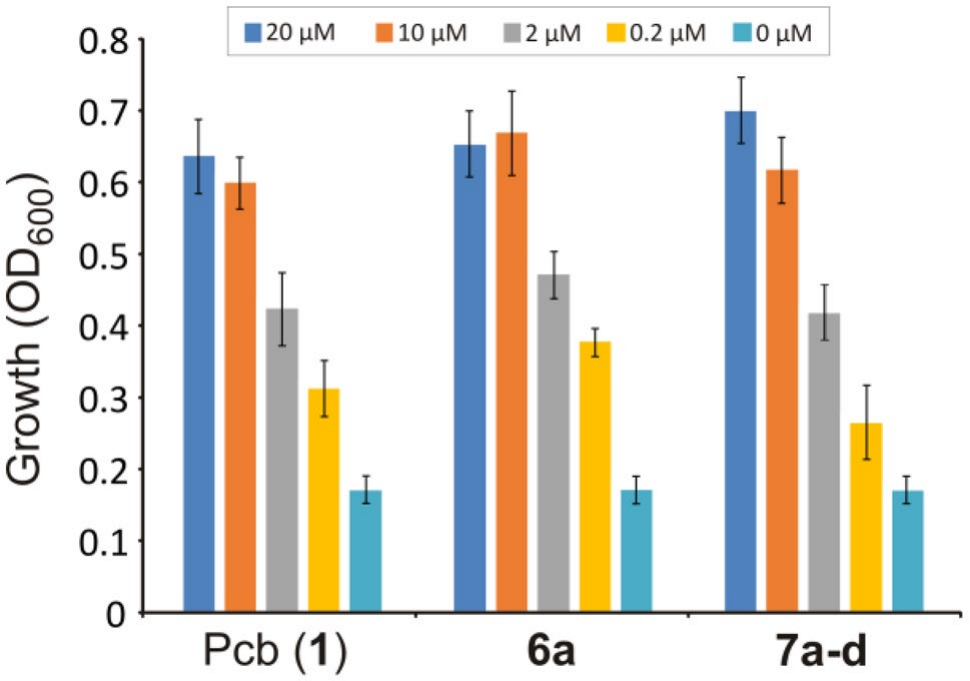
Utilization as iron source (maximum growth achieved) of Pcb (**1**) and the synthetic piscibactin analogues **6a** and **7** by *P. damselae* subsp*. piscicida* DI21 carrying a functional FrpA

Additionally, growth promotion assays against *Pdp* DI21 strains were used to study the biological activity of Pcb (**1**) and synthetic Pcb thiazole analogues **6** and **7** in these species (Fig. 8). The results showed that 13*S* piscibactin analogues are also internalized by *P. damselae* subsp. *piscicida* via FrpA_*Pdp*_, and also suggest that this transporter displays almost the same specificity for the synthetic 13*S* Pcb analogues **6** and **7** than for Pcb (**1**). All results together greatly suggest that the activity of piscibactin analogues synthesized here could be used not only to vectorize compounds against *V. anguillarum* but also against *Pdp* and those other bacterial species that carry *irp*-HPI like elements in their genome.

## Conclusion

The recognition of iron acquisition systems mediated by siderophores as relevant bacterial virulence factors and the fact that they are essential for the survival and growth of pathogens inside their hosts make them excellent candidates for the development of novel antimicrobial strategies. Pcb system is widespread among diverse marine bacterial pathogens and, consequently, it could be used as a target to prevent the occurrence of some infectious diseases. In this regard, it has been previously shown that FrpA_*Pdp*_, the piscibactin transporter of *P. damselae* subsp. *piscicida*, could be used as an immunogenic protein to formulate vaccines against fish photobacteriosis [33]. Alternatively, a different application could be based on the use of siderophore conjugates to vectorize antimicrobials following a Trojan horse approach [11] or to design fluorescent probes that could be used in the early diagnosis [34].

In this work, FrpA was characterized as the TonB-dependent transporter that mediates ferri-piscibactin uptake. Then, the evaluation of some synthetic Pcb analogues led to the definition of the siderophore chemical structures that enable ferri-piscibactin recognition by FrpA, its cognate outer membrane transporter. Our results showed that the presence of a thiazole ring instead of thiazoline scarcely affects the biological activity of piscibactin analogues. By contrast, the maintenance of the natural piscibactin configuration at position C-13 is crucial for Fe^3+^ chelation and, therefore, for the recognition of the ferri-siderophore by the cognate OMT. Our results also demonstrated that Pcb thiazole analogues can be internalized by *V. anguillarum* and by *Pdp* via FrpA_*Vang*_ and FrpA_*Pdp*_, respectively. Hence, it is predicted that Pcb analogues conjugates could be active for a large diversity of *irp*-HPI-carrying bacteria such as the fish pathogens *V. anguillarum* and *P. damselae* subsp. *piscicida*; bivalve pathogens including *V. ostreicida*, *V. neptunius*, and *V. coralliilyticus*; and also strains of *V. cholerae*. All together, these findings allowed us to propose a Pcb analogue as a good candidate to vectorize antimicrobial compounds, through the Trojan horse strategy, to develop novel compounds against bacterial fish diseases.

Future studies will allow us to test the use of different conjugates based on piscibactin to develop novel antimicrobials or other compounds with diverse biotechnological applications, that can be efficiently transported into *Vibrionaceae* bacterial cells using FrpA outer membrane transporter.

## Supplementary Information

Below is the link to the electronic supplementary material.

Supplementary file1 (PDF 218 KB) Strains and plasmids used (Table S1), and oligonucleotides used for construction of frpA defective mutants and for gene complementation (Table S2).
