# Supplementary material for: FrpA is the outer membrane piscibactin transporter in Vibrio anguillarum: structural elements in synthetic piscibactin analogues required for transport
Source: J Biol Inorg Chem. 2021 Nov 18;27(1):133–42. doi: 10.1007/s00775-021-01916-1 (PMC8840927; doi:10.1007/s00775-021-01916-1)
Supplement: Supplementary file 1 — Supplementary file1 (PDF 218 KB) Strains and plasmids used (Table S1), and oligonucleotides used for construction of frpA defective mutants and for gene complementation (Table S2). [file 775_2021_1916_MOESM1_ESM.pdf]

Supplementary Information

FrpA is the outer membrane piscibactin transporter in *Vibrio anguillarum*: structural elements in synthetic piscibactin analogues required for transport

Marta A. Lages[1], M. Carmen de la Fuente[2], Lucía Ageitos[2], Diana Martínez-Matamoros[2], Jaime Rodríguez[2]*, Miguel Balado[1]*, Carlos Jiménez[2]*, and Manuel L. Lemos[1]

[1] Departamento de Microbiología y Parasitología, Instituto de Acuicultura, Universidade de Santiago de Compostela, 15782 Santiago de Compostela, Spain
[2] Centro de Investigacións Científicas Avanzadas (CICA), Departamento de Química, Facultade de Ciencias, Universidade da Coruña, 15071 A Coruña, Spain

Table S1: Strains and plasmids used in this work.

Strain	Relevant characteristics	Source
V. anguillarum		
MB14	RV22 with in-frame deletion of *vabF* gene<br>RV22 Δ*vabF*	Balado *et. al.*, 2006[1]
MB67	RV22 with in-frame deletion of *vabD* gene<br>RV22 Δ*vabD*	Balado *et. al.*, 2008[2]
ML208	RV22 with in-frame deletion of *vabF* and *frpA* genes<br>RV22 Δ*vabF*Δ*frpA*	This study
ML210	RV22 with in-frame deletion of *vabD* and *frpA* genes<br>RV22 Δ*vabD*Δ*frpA*	This study
Photobacterium damselae* subsp. *piscicida		
DI21	Piscibactin producer strain	Toranzo *et. al.*[3]
E. coli		
DH5α	Cloning strain	Laboratory strain
S17-1-<br>*λpir*	RP4 (Km::Tn7, Tc::Mu-1) *pro-82 λpir recA1 end A1 thiE1*<br>*hsdR17 creC510*	Herrero et. al.[4]
**Plasmids**		
pWKS30	Low-copy number cloning vector, Amp^r	Wang and Kushner[5]
pNidKan	Suicide vector derived from pCVD442, Kan^r	Mouriño *et. al.*[6]
pML257	S17 λpir pSEVA 651 frpA comp	This study

[1] Balado, M.; Osorio, C. R.; Lemos, M. L. A gene cluster involved in the biosynthesis of vanchrobactin, a chromosome-encoded siderophore produced by *Vibrio anguillarum*. *Microbiology* **2006**, *152*, 3517–3528. doi: 10.1099/mic.0. 29298-0.

[2] Balado, M.; Osorio, C. R.; Lemos, M. L. Biosynthetic and regulatory elements involved in the production of the siderophore vanchrobactin in *Vibrio anguillarum*. *Microbiology* **2008**, *154*, 1400–1413. doi: 10.1099/mic.0.2008/ 016618-0.

[3] Toranzo, A. E.; Barreiro, S.; Casal, J. F.; Figueras, A.; Magariños, B.; Barja, J. L. Pasteurellosis in cultured gilthead seabream (*Sparus aurata*): first report in Spain. Aquaculture **1991**, *99*, 1–15. doi: 10.1016/0044-8486(91)90284-E.

[4] Herrero, M.; de Lorenzo, V.; and Timmis, K. N. Transposon vectors containing non-antibiotic resistance selection markers for cloning and stable chromosomal insertion of foreign genes in Gram-negative bacteria. *J. Bacteriol.* **1990**, *172*, 6557–6567. doi: 10.1128/jb.172.11.6557-6567.1990.

[5] Wang, R. F.; Kushner, S. R. Construction of versatile low-copy-number vectors for cloning, sequencing and gene expression in *Escherichia coli*. Gene **1991**, *100*, 195–199. doi: 10.1016/0378-1119(91)90366-J.

[6] Mourino, S.; Osorio, C. R.; Lemos, M. L. Characterization of heme uptake cluster genes in the fish pathogen *Vibrio anguillarum*. *J. Bacteriol.* **2004**, *186*, 6159–6167. doi: 10.1128/JB.186.18.6159-6167.2004

Table S2: Oligonucleotides used for construction of *frpA*-defective mutants by allelic exchange and oligonucleotides complementation. Underlined sequences denote restriction sites.

Oligonucleotide	Sequence (5' → 3')	Amplicon Size (bp)
***frpA* mutant construction**		
1_FrpAang_XbaI	CGCTCTAGAGGCAGCACTCAACAACAAGG	750
2_FrpAang_BamHI	GCGGGATCCGAGTGCGAAAGCGGTTGAAG	
3_FrpAang_BamHI	CCGGGATCCAACCTATTCGTTCGCAATGC	750
4_FrpAang_4_XhoI	GCGCTCGAGGCTTTCAGAGATGGATGGTG	
***frpA* complementation**		
1_FrpAang_comp_F_XbaI	CGCTCTAGACTCAGCGCAGAAATCTACAC	2570
2_FrpAang_comp_R_BamHI	GGCGGATCCAGAGACGCCTTCTGAACGCA	
